# PEG–PLGA nanoparticles for encapsulating ciprofloxacin

**DOI:** 10.1038/s41598-023-27500-y

**Published:** 2023-01-06

**Authors:** Natsorn Watcharadulyarat, Monthira Rattanatayarom, Nisarat Ruangsawasdi, Nisa Patikarnmonthon

**Affiliations:** 1 Department of Biotechnology, Faculty of Science, Mahidol University, Bangkok, 10400 Thailand; 2 Department of Pharmacology, Faculty of Dentistry, Mahidol University, Bangkok, 10400 Thailand; 3 Mahidol University-Osaka University Collaborative Research Center for Bioscience and Biotechnology, Faculty of Science, Mahidol University, Bangkok, 10400 Thailand

**Keywords:** Nanoparticles, Root canal treatment

## Abstract

Antibiotic medications have been found to hinder the success of regenerative endodontic treatment due to the rapid degradation of the drug, and the acidic nature of ciprofloxacin (CIP) can be harmful to stem cells of the apical papilla (SCAPs), the cells responsible for regeneration. In this study, a nanocarrier system was used for controlled drug release for longer drug activity and less cytotoxicity to the cells. CIP was loaded in poly (ethylene glycol) methyl ether-*block*-poly (lactide-*co*-glycolide) (PEG–PLGA) nanoparticles (NPs) with an ion-pairing agent. The NPs demonstrated a monodispersed spherical morphology with a mean diameter of 120.7 ± 0.43 nm. The encapsulation efficiency of the CIP-loaded PEG–PLGA NPs was 63.26 ± 9.24%, and the loading content was 7.75 ± 1.13%. Sustained CIP release was achieved over 168 h and confirmed with theoretical kinetic models. Enhanced NP bactericidal activity was observed against *Enterococcus faecalis*. Additionally, CIP-loaded PEG–PLGA NPs had a low cytotoxic effect on SCAPs. These results suggest the use of a nanocarrier system to prolong the antibiotic activity, provide a sterile environment, and prevent reinfection by the bacteria remaining in the root canal during regenerative endodontic treatment.

## Introduction

In regenerative endodontic treatment, complete removal of the microbial infection in the dental pulp is required for successful root canal therapy^1^. Topical antibiotic delivery into the root canals provides several advantages, such as higher drug concentration, specificity, and preventing systemic adverse effects^2^. Ciprofloxacin, a component of the triple antibiotic pastes applied as a root canal medicament^3^, is a broad-spectrum antibiotic targeting *E. faecalis*, which commonly leads to endodontic failure. However, the use of ciprofloxacin has demonstrated limitations for regenerative endodontics. The acidity of the hydrophilic ciprofloxacin inhibited stem cell proliferation and differentiation when it was loaded at high concentration^4^. In addition, the antibiotic paste medicament rapidly degrades in the root canal, which can result in lowering the antibiotic concentration below its effective dose^2^. Therefore, developing a novel topical antibiotic delivery system is essential for prolonging the drug’s activity and being compatible with stem cells.

A scaffold loaded with ciprofloxacin was developed to control the release rate and prolong the drug’s activity. However, a burst-release was observed in several reports^5,6^ due to the hydrophilicity of the hydrogel, which could not maintain and stabilize the ciprofloxacin^7–9^.

In contrast, loading a hydrophilic drug into a hydrophobic matrix resulted in extended release^10^. Antibiotics nanoencapsulation has become a promising strategy to enhance the efficacy and minimize the adverse effects associated with conventional drug application^11^. A nanocarrier prevents early drug degradation from enzymatic attack and reduces the toxicity of the drug due to sustained drug release^11,12^. Due to their small size, nanocarriers provide a large surface area-to-mass ratio resulting in greater interaction and better permeation in bacteria cells, as well as increasing the drug’s retention time at the infection site^13^. Compared with other nanoparticle types, polymeric nanoparticles improved the antibiotic’s pharmacokinetic and pharmacodynamic profiles, lowered the required dose, reduced the drugs’ side effects, and decreased their cytotoxic effect on stem cells^14^.

Encapsulating ciprofloxacin in the hydrophobic part of the polymeric nanoparticles could be an effective approach to control drug release and maintain drug activity^15^. Poly (ethylene glycol) methyl ether-*block*-poly (lactide-*co*-glycolide) or PEG–PLGA is an amphiphilic polymer, which self-assembles in the aqueous phase and spontaneously generates nanoparticles^16^. Based on their excellent biodegradability and biocompatibility, PEG and PLGA have been approved by the US FDA^17^. Although polymeric nanoparticles are a good candidate for enhancing the antibiotic’s efficiency and maintaining drug release, encapsulating the hydrophilic ciprofloxacin in nanoparticles is challenging due to its high diffusion rate^18^. Therefore, ion complexation due to the electrostatic interaction between ciprofloxacin and an ion pairing agent, such as 2-ethylbutyl cyanoacrylate (EBCA)^19^, sodium deoxycholate^7^, oleic acid^20^, or linoleic acid-conjugated chitosan^21^, has been proposed to improve the drug loading efficiency in nanoparticles. The solid-in-oil-in-water (S/O/W) ion pairing method^22,23^ using dextran sulfate as the ion pairing agent was introduced to achieve high drug encapsulation efficiency.

Various types of nanoparticles have demonstrated their potential in endodontic treatment, especially as an intracanal medicament. Copper nanoparticles (CuNPs) were proposed to use as a disinfectant in endodontic treatment. They had an antimicrobial effect against a bacterial biofilm^24^. Chitosan nanoparticles were employed as an antimicrobial against endodontic pathogens, including *E. faecalis,* and did not have a cytotoxic effect on mouse fibroblast cells^25^. Another study developed chitosan-coated PLGA nanoparticles to encapsulate ciprofloxacin for preventing root canal infection^26^.

Although several studies used PLGA to encapsulate ciprofloxacin^20,26,27^, to the best of our knowledge, encapsulating ciprofloxacin in PEG–PLGA nanoparticles has not been reported. Here, ciprofloxacin-loaded PEG–PLGA nanoparticles prepared using the S/O/W ion pairing method were evaluated for their potential as an antibiotic nanocarrier due to their physical properties and high drug encapsulation efficiency. The nanoparticles’ sustained drug release was also determined by the release kinetics. The CIP-loaded PEG–PLGA nanoparticles were evaluated for their antibacterial activity against a dental pathogen, *E. faecalis*, and their cytotoxic effect on human mesenchymal stem cells from the apical papilla (SCAPs).

## Materials and methods

### Materials

Poly (ethylene glycol) methyl ether-*block*-poly(lactide-*co*-glycolide) with PEG (average MW of 2000 g/mol), PLGA (average MW of 11,500 g/mol) (lactide: glycolide = 50:50), and dextran sulfate sodium salt from *Leuconostoc* spp. with a molecular weight more than 500,000 g/mol were purchased from Sigma–Aldrich, Germany. Phosphotungstic acid hydrate was purchased from Sigma–Aldrich, Japan. Ciprofloxacin HCl (CIP) was purchased from Cayman Chemical Company, USA. Acetone was purchased from RCI Labscan, Thailand. Brain heart infusion broth (BHI) was obtained from Difco Laboratories, Detroit, Michigan, USA. Alpha minimum essential medium (αMEM), Dulbecco's Modified Eagle Medium (DMEM), fetal bovine serum, and penicillin/streptomycin were obtained from Gibco, Life Technologies, Grand Island, NY, USA. Analytical grade solvents were purchased from Sigma–Aldrich and used as received.

*Enterococcus faecalis* (ATCC^®^ 19,433™) was used in this study. The SCAPs were obtained from Dr. Ruangsawasdi Nisarat at the Faculty of Dentistry, Mahidol University, Thailand after approval by the Institutional Review Board of the Human Ethics Committee of the Faculty of Dentistry, Mahidol University (COE. No. MU-DT/PY-IRB 2022/010.0202). The SCAPs were characterized in a previous report^28^.

### Methods

#### CIP-loaded PEG–PLGA nanoparticle preparation

The solid-in-oil-in-water (S/O/W) ion pairing method was modified from a prior study^22^. PEG–PLGA was dissolved in acetone at a concentration of 20 mg/ml. 0.07 ml CIP prepared in distilled water at final concentrations of 10, 20, and 35 mg/ml (equivalent to the initial weight of CIP of 0.7, 1.4, and 2.45 mg, respectively) was added to 1 ml of the polymer solution followed by 0.03 ml 80 mg/ml dextran sulfate in distilled water. The mixture was poured into 6 ml distilled water with magnetic stirring (C-MAG HS7, IKA, Germany). The nanoparticle suspension was dialyzed using a dialysis membrane with a 6000–8000 MW cut-off of (Spectra/Por, Thomas Scientific, USA) against distilled water at 25 °C to remove the organic solvent and free CIP. The distilled water was changed every 3 h for 3 times.

#### CIP-loaded PEG–PLGA nanoparticle characterization

A dynamic light scattering (DLS) particle size analyzer (Malvern Zeta Nanosizer ZS, Malvern Instruments Ltd, UK). was used to determine the CIP-loaded PEG–PLGA NP hydrodynamic size (Z-average size), polydispersity index (PdI), and ζ-potential. The nanoparticles were tenfold diluted in filtered distilled water. A disposable Folded Capillary cell (Malvern DTS1070) was used to determine the ζ-potential. The PEG–PLGA nanoparticles morphology was observed using transmission electron microscopy (TEM) (HT-7700, Hitachi High-Tech Corporation, Japan). The sample was prepared on a carbon-coated copper grid and negatively stained with a 2.0%w/v phosphotungstic acid solution. The imaging was performed at magnification of 15,000 and 30,000 × with 100 kV acceleration voltage.

#### Drug encapsulation determination

One ml CIP-loaded PEG–PLGA NPs were lyophilized (Freeze One6 Plus, LABCONCO, USA) and re-dissolved in the equivalent volume of DMSO with 0.05 N HCl. The solution was then 20-fold diluted with distilled water. The CIP absorbance was detected at a wavelength of 275 nm using a microplate reader (MULTISKAN GO, Thermo Scientific). The encapsulation efficiency (%EE) and loading content (%LC) were determined according to the following equations.

$$\% {\text{Encapsulation}}\;{\text{efficiency}} = \frac{{{\text{Weight}}\;{\text{of}}\;{\text{drug}}\;{\text{in}}\;{\text{nanoparticles}}}}{{{\text{Initial}}\;{\text{weight}}\;{\text{of}}\;{\text{drug}}}} \times 100$$

$$\% {\text{Loading}}\;{\text{content}} = \frac{{{\text{Weight}}\;{\text{of}}\;{\text{drug}}\;{\text{in}}\;{\text{nanoparticles}}}}{{{\text{Total}}\;{\text{weight}}\;{\text{of}}\;{\text{nanoparticles}}}} \times 100$$

#### In vitro release profile of CIP-loaded PEG–PLGA NPs

The in vitro drug release was determined using the dialysis method. CIP-loaded PEG–PLGA NPs in a 10%w/v sucrose solution were lyophilized. Three ml CIP-loaded PEG–PLGA NPs and free CIP in sterile distilled water at the equivalent CIP concentration (0.3 mg/ml) were placed in a dialysis membrane and dialyzed against a 20 ml sterile tris-buffer saline solution (TBS), pH 7.4. The experiment was conducted in a closed and dark environment, preventing the evaporation of the release media and the possibility of microbial contamination. The samples were shaken at 100 rpm at 37 °C. After 0, 2, 4, 6, 8, 24, and 168 h of incubation, 20 ml release medium was collected and stored at − 20 °C. The old medium was replaced with 20 ml of sterile TBS, pH 7.4. The concentration of ciprofloxacin released in the solution was measured using a spectrophotometer at a wavelength of 275 nm. The release kinetics were evaluated during the first 60 min of drug release. Three ml CIP-loaded PEG–PLGA NPs was dialyzed against 20 ml of sterile TBS, pH 7.4. 0.5 ml of the release medium was taken at 15, 30, 45, and 60 min and replaced with 0.5 ml fresh TBS, pH 7.4. The concentration of CIP released in the medium was measured using a spectrophotometer. The data were evaluated using theoretical models comprising zero order^29^, first order^29^, Higuchi^30^, Korsmeyer-Peppas^31^, and Hixson-Crowell^32^. Nonlinear regression (curve fit) was performed using GraphPad Prism version 9.1.2 for Windows (GraphPad Software, La Jolla California USA, www.graphpad.com).

#### Antibacterial activity of the CIP-loaded PEG–PLGA nanoparticles

The antibacterial activity of the CIP-loaded PEG–PLGA nanoparticles was analyzed as previously described^5^. Briefly, the minimal inhibitory concentration (MIC) and minimal bactericidal concentration (MBC) of the CIP-loaded PEG–PLGA NPs were determined against *E. faecalis*. The bacteria were inoculated in BHI broth and incubated at 37 °C overnight without shaking. The bacterial culture was diluted with BHI broth and the optical density (WPA CO 8000 Biowave Cell Density Meter, Biochrom Ltd., UK) was measured at 600 nm. The optical density was adjusted to reach the McFarland 0.5 standard solution (equivalent to 1.5 × 10^8^ CFU/ml). The bacterial culture was further diluted 100-fold with BHI broth to obtain a final concentration of ~ 1 × 10^6^ CFU/ml. The microbial suspension was treated with the CIP-loaded PEG–PLGA NPs, free CIP, and empty PEG–PLGA NPs for 24 h at 37 °C. The amount of CIP in the free CIP and the CIP-loaded PEG–PLGA NPs was ranging between 0.5 and 64 µg/ml. The concentration of CIP in the CIP-loaded PEG–PLGA NPs was calculated based on the %EE. The empty PEG–PLGA NPs at a corresponding amount of CIP-loaded PEG–PLGA NPs were used as a control experiment to observe the effect of empty PEG–PLGA NPs on the bacterial cells. The lowest concentration of CIP with no turbidity on the plate well was considered the MIC. Next, an 8 µl clear solution from the plate well was dropped on the BHI agar plate and the plate was incubated for 24 h at 37 °C. The minimum concentration of the CIP with no colonies present on the BHI agar was considered the MBC.

#### Cytotoxicity of the CIP-loaded PEG–PLGA nanoparticles

SCAPs were cultured in αMEM supplemented with 10% fetal bovine serum, 100 U/ml Penicillin, and 100 µM/ml Streptomycin. SCAPs were incubated at 37 °C with 5% CO_2_. The culture medium was changed every two days until confluence was reached. The cytotoxicity of the CIP-loaded PEG–PLGA nanoparticles was investigated using an MTT assay. 1 ml CIP-loaded PEG–PLGA NP/10%w/v sucrose suspension was lyophilized and then resuspended with 0.1 ml sterile distilled water. The samples (CIP-loaded PEG–PLGA NPs, or free CIP) were diluted in culture media to obtain a final concentration of CIP at 5–100 µg/ml. SCAPs (10,000 cells/well) were seeded in a 96-well plate and cultured in complete α-MEM and incubated at 37 °C with 5% CO_2_ for 24 h. The cells were treated with the samples for 24 h before determining the SCAP cell viability using the MTT assay^33^. Briefly, 0.5 mg/ml MTT solution in DMEM media was added to each well and incubated in the dark for 2 h at 37 °C, with 5% CO_2_. The precipitated formazan crystals were dissolved in 200 µl DMSO and the absorbance was measured at 570 nm and 690 nm using a microplate reader. The percent cell viability of the treated SCAPs was compared with the untreated SCAPs as a control.

#### Statistical analysis

The statistical analysis was performed using GraphPad Prism version 9.1.2. The results were reported as mean ± standard deviation. A two-sided, unpaired t-test was used to evaluate the difference between two groups, while analysis of variance (ANOVA) followed by Tukey’s multiple comparisons test was performed to determine the treatment effect from more than 3 groups. Significant differences are represented as *, **, ***, **** for *P* < 0.05, 0.01, 0.001, and 0.0001, respectively.

## Results and discussion

### Preparation and characterization of the CIP-loaded PEG–PLGA nanoparticles

**Figure 1:**
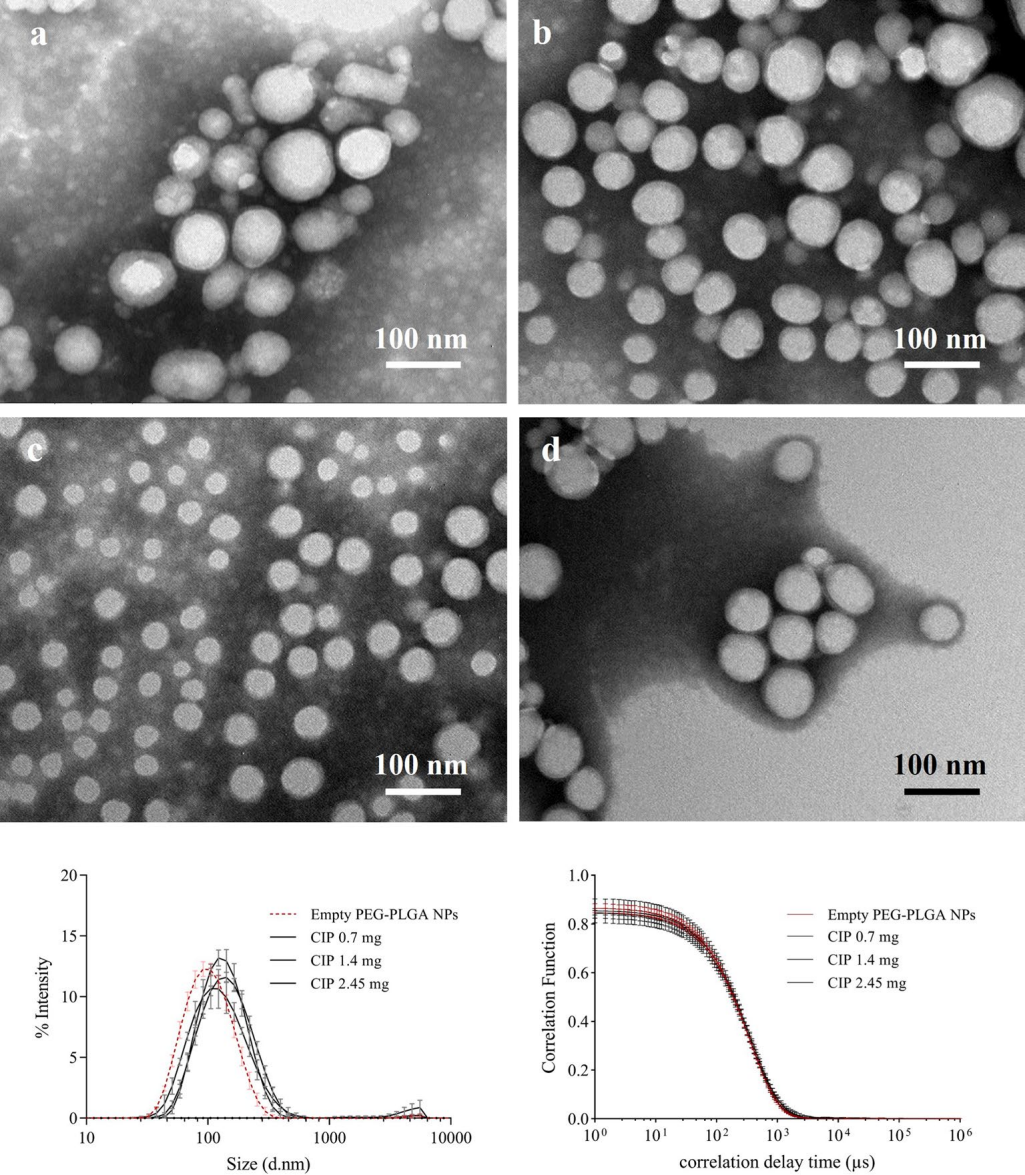
Electron micrographs of the empty PEG–PLGA NPs (**a**), CIP-loaded PEG–PLGA NPs at CIP initial weight of 0.7 mg (**b**), 1.40 mg (**c**), and 2.45 mg (**d**) observed by TEM. The images were taken at 40,000 × magnification. The plots represent the size distribution by the intensity and correlation function of the samples, the error bars in the plots refer to the standard deviation (s.d.) obtained from three independent experiments*.*

Empty PEG–PLGA NPs and CIP-loaded PEG–PLGA NPs were prepared using the S/O/W ion pairing method. The obtained nanoparticles were found to be small and homogeneous as seen in the size distribution graph (Fig. 1). The size of the empty PEG–PLGA NPs (92.8 ± 2.27 nm) was significantly smaller than the CIP-loaded PEG–PLGA NPs, which ranged between 110 and 120 nm (Table 1). The morphology of the PEG–PLGA NPs was observed by TEM (Fig. 1A–D). The PEG–PLGA NPs were spherical with a similar mean diameter both with and without CIP. These findings confirmed that the size and shape of nanoparticles can be controlled when prepared using the S/O/W ion pairing method.

**Table 1:** Characteristics of the PEG–PLGA nanoparticles prepared using the S/O/W ion pairing method.

CIP initial weight (mg)	Z-average (d.nm)	PdI	ζ-potential (mV)	%EE	%LC
0.00	92.8 ± 2.27	0.184 ± 0.024	− 49.6 ± 11.42	–	–
0.70	118.9 ± 3.79***	0.190 ± 0.011	− 41.3 ± 5.62	27.63 ± 1.72	0.97 ± 0.06
1.40	115.5 ± 6.09***	0.215 ± 0.024	− 41.0 ± 5.87	32.23 ± 1.78	2.30 ± 0.12
2.45	120.7 ± 0.43****	0.172 ± 0.013	− 40.0 ± 3.60	63.26 ± 9.24	7.75 ± 1.13

*** and **** refer to the significant difference between the CIP-loaded PEG–PLGA nanoparticles and empty PEG–PLGA nanoparticles (***P < 0.001 and ****P < 0.0001), %EE: encapsulation efficiency, %LC: loading content.

The ζ-potential of the CIP-loaded PEG–PLGA NPs demonstrated a strong negative charge (-40.00 mV) on the surface of the nanoparticles due to the excess amount of sulfate groups in dextran sulfate (Table 1). These results indicated that the CIP-loaded PEG–PLGA NPs were highly stable because the ζ-potential values were lower than − 30 mV leading to electric stabilization. Their negative charges tend to repel each other and prevent them from self-aggregating and flocculation^34^. Interestingly, the ζ-potential of the empty PEG–PLGA NPs was approximately − 50.0 mV, which was lower than the CIP-loaded PEG–PLGA NPs. The increased ζ-potential is due to the neutralization of the negative charge by a cationic molecule, such as ciprofloxacin^23^.

The %EE and %LC of the CIP-loaded PEG–PLGA NPs increased in a dose-dependent manner (Table 1). CIP at the initial weight of 2.45 mg provided the highest %EE and %LC, which was significantly higher than CIP at the initial weight of 1.4 mg (%EE: *P* = 0.0011, %LC: *P* = 0.0001) and 0.7 mg (%EE: *P* = 0.0005, %LC: *P* < 0.0001). Thus, the CIP-loaded PEG–PLGA NPs at the CIP initial weight of 2.45 mg, which had the highest EE, were used for further experiments.

The CIP-loaded PEG–PLGA NPs were successfully prepared using the S/O/W ion pairing method. This method exhibits some advantages similar to the conventional nanoprecipitation method, which are simple, low energy requirement, controllable, reproducible, and up-scalable. Furthermore, it can overcome the low encapsulation efficiency of the nanoprecipitation method^35^. The successful encapsulation could result from adding dextran sulfate. Dextran sulfate has been used in protein-based medicine or with multivalent peptides to facilitate the encapsulation process^36^. It plays an important role as an ion-pairing agent to decrease the water solubility of hydrophilic ciprofloxacin.

**Figure 2:**
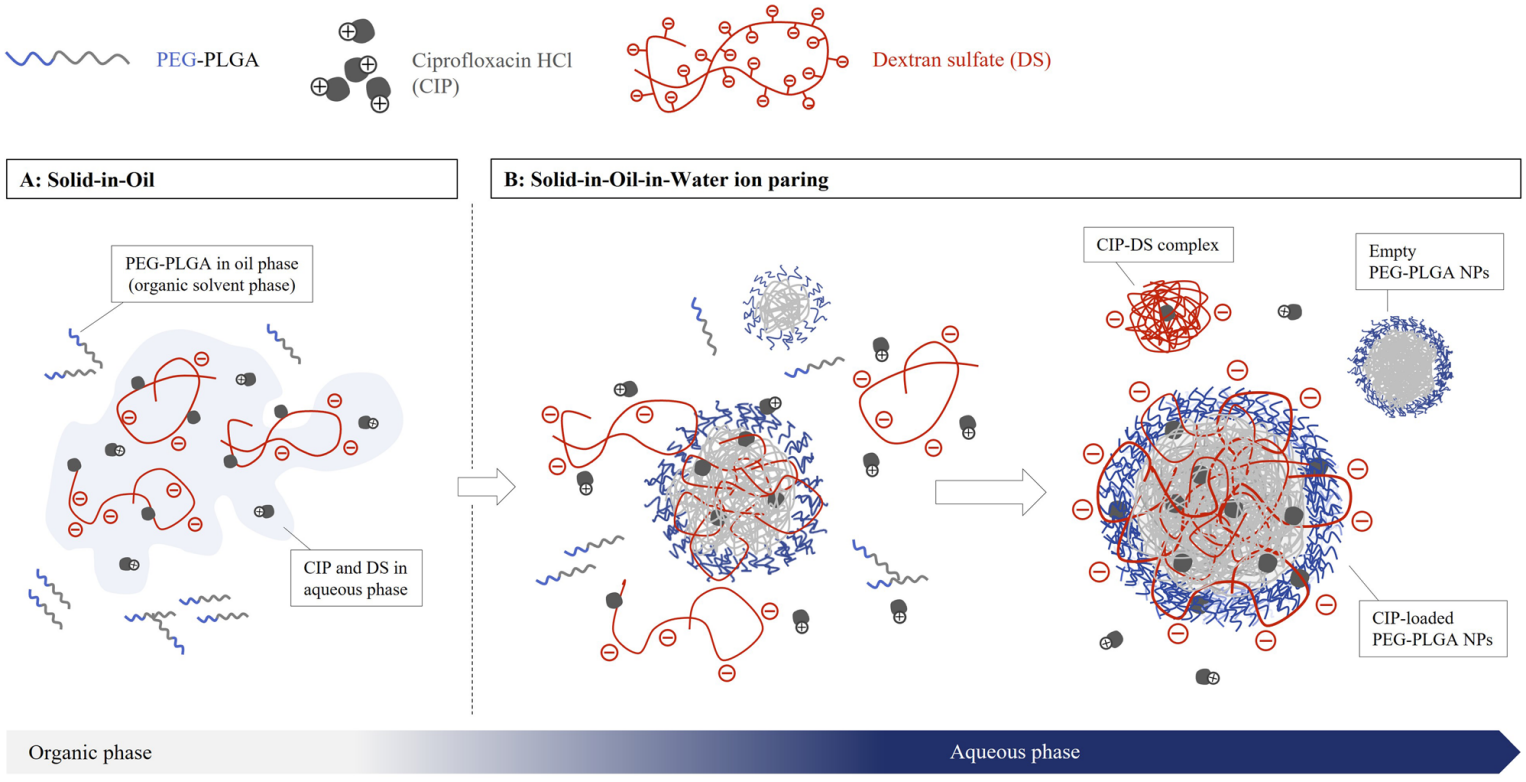
Illustration of CIP-loaded PEG–PLGA NP formation via the S/O/W ion paring process (created from Microsoft 365 PowerPoint version 2211, www.microsoft.com), solid-in-oil (S/O) process (**A**); CIP was added into the PEG–PLGA suspension in acetone, followed by the dextran sulfate solution. S/O/W process (**B**); the mixture (**A**) was poured into distilled water to form nanoparticles via a self-assembly process.

The CIP-loaded PEG–PLGA NPs were larger than the empty PEG–PLGA NPs (*P* = 0.0001 for CIP 0.70 mg, *P* = 0.0004 for CIP 1.40 mg, and *P* < 0.0001 for CIP 2.45 mg) due to the presence of CIP in the inner layer of the nanoparticles. However, the sizes did not depend on the drug content, suggesting that the additional CIP could interact with the excess amount of dextran sulfate via electrostatic force and were located on the surface of the nanoparticles^23^. The ionic interaction can occur randomly between a protonated amine group of ciprofloxacin and the sulfate group anions in dextran sulfate (see supplementary data, Fig. S1), which was explained in a previous report^7^. Figure 2 presents the principle of nanoparticle formation and demonstrates the electrostatic interaction between CIP and dextran sulfate. It was hypothesized that CIP can form a complex with dextran sulfate during various steps. The CIP and dextran sulfate complexes formed prior to polymer self-assembly (Fig. 2A) could be trapped inside the nanoparticles, while the complexes that form after the self-assembly of PEG–PLGA (Fig. 2B**)** can locate on the surface of nanoparticles^23^. Although dextran sulfate was demonstrated to increase the encapsulation efficiency of CIP in this work, amphiphilic polymers (PEG–PLGA in this case) also play an important role in determining NP size, because only CIP-dextran sulfate complexes with a wide range in size and agglomeration were readily observed (see supplementary data, Fig. S1). The size of the CIP-dextran sulfate complex was ~ 388.47 nm with a PdI equal to 0.556.

### In vitro release profile of CIP-loaded PEG–PLGA NPs

**Figure 3:**
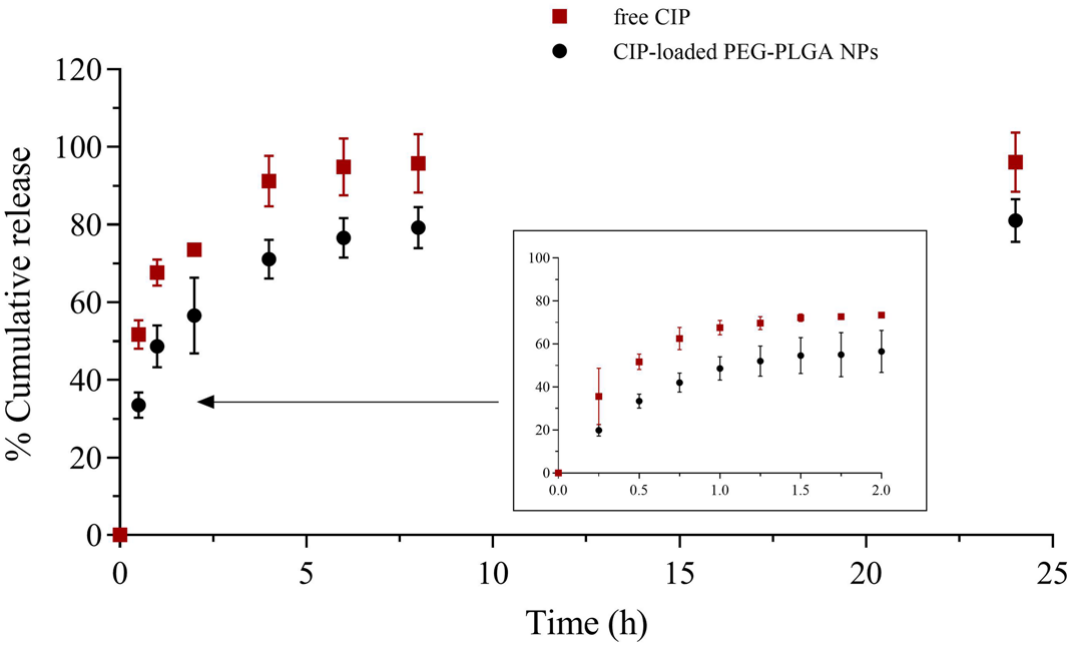
The cumulative release of free CIP (■) and CIP-loaded PEG–PLGA NPs (●) in tris-buffer saline solution, pH 7.4 over 24 h. The samples were shaken at 100 rpm at 37 °C, in the dark. The error bars in the plots represent the standard deviation obtained from three independent experiments of each sample.

The CIP release of the CIP-loaded PEG–PLGA NPs compared with free CIP was determined. CIP was stable during the experiment as previously reported^5^. The results are shown as the cumulative %drug release versus time (h). The drug release profile during the first 24 h is presented in Fig. 3. After the first 4 h, CIP in the free form demonstrated 91.22 ± 6.55% drug diffusion. The CIP-loaded PEG–PLGA NPs showed a significant improvement in controlled release (*P* < 0.001 at 1, 2, and 6 h and *P* < 0.01 at 8 and 24 h). CIP gradually diffused from the PEG–PLGA NPs at 71.09 ± 5.00% after 4 h and peaked at 83.97 ± 5.67% after 168 h (see supplementary data, Fig. S2).

The results suggested that the controlled release was improved when using a nanocarrier system compared with free CIP. Normally, the drug release is dominated by certain mechanisms, e.g., diffusion, dissolution, and the swelling of a matrix^37,38^. However, de-complexation between CIP and dextran sulfate could be an additional mechanism that decelerates the release of the hydrophilic drug^22^, even though the dissolution rate of CIP is high due to its hydrophilicity^39^. The counterion competition occurs when salt is presented in the solution. Sodium ions (Na^+^) in the buffer can act as a cation to replace the CIP molecules on the dextran sulfate branch. The rapid release can be due to CIP on the surface of the nanoparticles, while the sustained release was obtained from CIP in the inner phase of the NPs, which is more challenging to be released^36^. The CIP release also correlates with the PLGA degradation rate. PLGA with a 50:50 ratio of lactide: glycolide tends to degrade within 2 weeks in an aqueous solution^40^. In clinical use, the CIP-loaded PEG–PLGA NPs will be entrapped in scaffolds as a combined platform to provide the advantages of maintaining a sterile environment and inducing tissue regeneration^20^. The scaffold could delay the degradation rate of PLGA by reducing the exposure of the nanoparticles to an aqueous environment. However, the incorporation with scaffolds and in vivo release should be further investigated to address these hypotheses in more detail.

The release kinetics were investigated to observe the release behavior of CIP from the PEG–PLGA NPs during the first 60 min (Table 2). The plots are seen in the supplementary data (Fig. S3). Although the R^2^ values of the zero order, first order, and Higuchi models were over 0.9, the release of CIP from PEG–PLGA NPs had the best fit with the Higuchi model due to the highest R^2^ values (0.9617), which was similar to a previous report^26^. The Higuchi model described the release of a drug from a thin film into the skin, assuming that the perfect sink condition is provided, the diffusion coefficient is constant, and the matrix is a non-swelling barrier^30,41^. However, an additional mathematical model was performed in parallel due to the limitations of the Higuchi model, for example, the release kinetics of the Higuchi model is based on one dimension and the diffusion coefficient of the drug is changeable^41^. Therefore, the Korsmeyer-Peppas model was also used. This model is a simple mathematical model derived from the power law^31^ describing drug release behavior from a polymeric system. The release exponent (n) indicates the drug release mechanism when the cumulative drug release is less than 60%. In the case of spherical NPs, n ≤ 0.43 can be assumed as Fickian diffusion, while 0.43 < n ≤ 0.85 is non-Fickian diffusion (anomalous transport)^42^. The release exponent of the CIP-loaded PEG–PLGA NPs (Table 2) was 0.6583, indicating that CIP tends to be released from the nanoparticles via drug diffusion, and the relaxation of the polymer particles^42^.

**Table 2:** The theoretical kinetic evaluation of the drug release profile of the CIP-loaded PEG–PLGA NP.

No	Model name	Simplified Equation	R^2^	n
1	Zero order	Q_t_ = Q_0_ + K_0_t	0.9240	–
2	First order	ln (100 – Q_t_) = ln (100—Q_0_) + K_1_t	0.9360	–
3	Higuchi	Q = K_H_(t^1/2^), when Q ≤ 60%	0.9617	–
4	Hixson-Crowell	(W_0_)^1/3^—(W_t_)^1/3^ = K_HC_t	0.8369	–
5	Korsmeyer-Peppas	Log (Q) = Log K_KP_ + n (Log t), when Q ≤ 60%	0.9296	0.6583

Q_t_ = % drug release at time t, Q_0_ = Initial concentration of the drug at t = 0, K_x_ = Drug release rate constant, n = Drug release exponent.

### Antibacterial activity of the CIP-loaded PEG–PLGA nanoparticles

The antibacterial activity of the CIP-loaded PEG–PLGA NPs and empty PEG–PLGA NPs against the oral pathogen, *E. faecalis*, is shown in Table 3. The MIC and MBC values of free CIP were 1.0 and 4.0 µg/ml, respectively. The results were comparable with the antimicrobial susceptibility test of ciprofloxacin against *E. faecalis* in the previous report where the MIC_90_ of ciprofloxacin ranged from 0.06 to 1 µg/ml^43^. However, 74 strains of *E. faecalis* presented high MBC_90%_ values (≥ 64 µg/ml)^44^, which were higher than the results obtained in this study. The differences might be due to many factors, such as bacterial strain, media, and using commercial drugs. The MIC of CIP-loaded PEG–PLGA NPs in this study was not significantly different when compared to the free drug, suggesting that CIP encapsulated in PEG–PLGA NPs provides similar growth inhibitory activity as the free CIP (*P* = 0.4347). In addition, the inhibitory effect on bacterial growth was not observed when using the empty PEG–PLGA NPs. These results indicate that the polymeric nanoparticles did not affect CIP’s antibacterial activity.

**Table 3:** The antimicrobial susceptibility test of the nanoparticles against *E. faecalis.*

Sample	MIC (µg/ml)	MBC (µg/ml)
Ciprofloxacin	1.00	4.00
PEG–PLGA NPs	ND	ND
CIP-loaded PEG–PLGA NPs	1.07 ± 0.19	2.83 ± 1.04**

*MIC* Minimum Inhibitory Concentration of CIP (µg/ml), *MBC* Minimum Bactericidal Concentration of CIP (µg/ml), *ND* Not Detected, the results are represented as mean ± standard deviation (n = 9), ** refers to the significant difference between the CIP-loaded PEG–PLGA NPs and free CIP (**P < 0.01).

In the previous report, the CIP-loaded solid lipid nanoparticles showed a greater inhibitory effect on *E. faecalis, Staphylococcus aureus, and Pseudomonas aeruginosa* when compared with the free CIP, due to the presence of triethylamine which enhances the lipophilicity of ciprofloxacin^45^. Other systems such as CIP with sodium deoxycholate^7^ and CIP in chitosan nanoparticles^46,47^ also could reduce the MIC of CIP. Meanwhile, the MIC and MBC of the CIP-loaded polyethylbutylcyanoacrylate (PEBCA) nanoparticles were similar to that of the free CIP when tested with *Salmonella enterica*^19^. Even though an inhibitory effect of CIP-loaded PEG–PLGA NPs in this work was not different from the free CIP, the bactericidal activity of the CIP-loaded PEG–PLGA NPs was enhanced as the MBC value was significantly lower than the MBC of free CIP (*P* = 0.0046). The results revealed that the polymeric nanoparticles protected CIP from chemical interaction with the surrounding environment, thus, the drug’s efficiency is maintained^48^. In addition, ion complexation improved bacterial cell membrane permeation by increasing the lipophilicity (Log P) of CIP^49^.

### Cytotoxicity of the CIP-loaded PEG–PLGA nanoparticles

**Figure 4:**
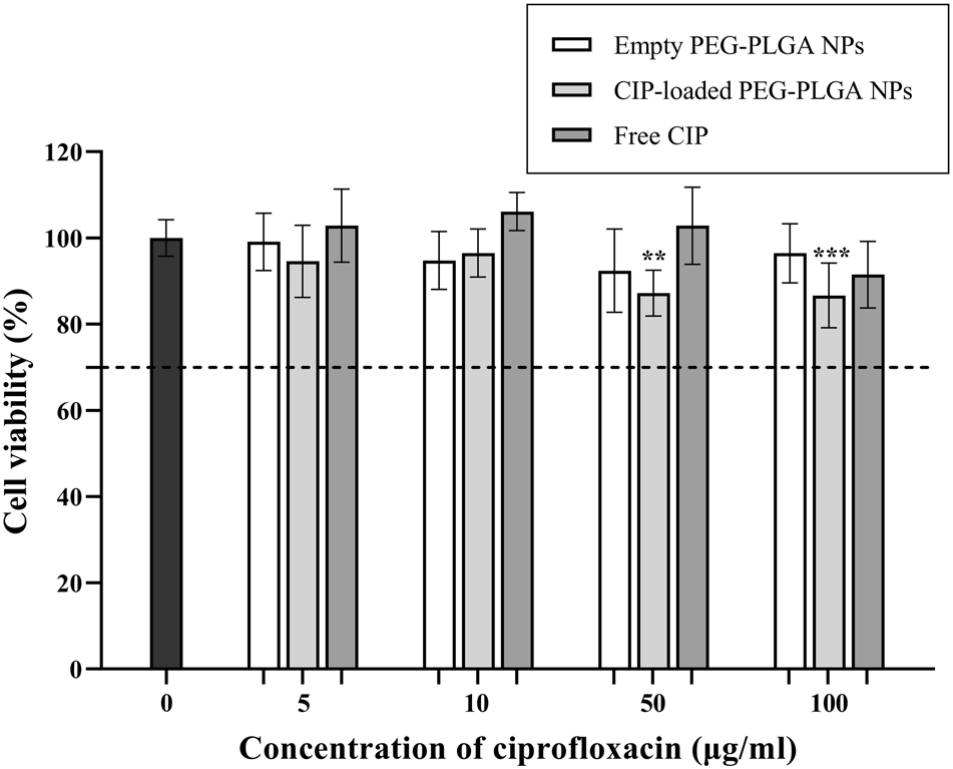
SCAP viability after being treated with empty PEG–PLGA NPs, CIP-loaded PEG–PLGA NPs, and free CIP for 24 h, the error bars represent the standard deviation from three independent experiments.

Stem cells from the apical papilla (SCAPs) isolated from the apical root of human immature permanent teeth are commonly used in endodontic regenerative treatment^50^. Therefore, the effect of the CIP-loaded PEG–PLGA NPs on SCAP viability was evaluated to assure the biocompatibility of the nanoparticles with SCAPs. Here, SCAPs were treated with empty PEG–PLGA NPs, CIP-loaded PEG–PLGA NPs, and free CIP for 24 h. SCAP viability was observed using an MTT assay^33^. The results demonstrated that the PEG–PLGA NPs at all tested concentrations were not cytotoxic to SCAPs (Fig. 4**)**. The SCAP viability was similar to that of the untreated cells (*P* > 0.05). The effect of free CIP (5–100 μg/ml) on SCAP viability was also investigated (Fig. 4).

The SCAP viability was 91.50% when treated with 100 μg/ml CIP, which was interpreted as being non-cytotoxic to the cells. A previous report demonstrated that 1 mg/ml antibiotics paste (minocycline, metronidazole, ciprofloxacin) decreased SCAP viability to 58% in 3 d^4^. Therefore, it was reasonable that 5–100 μg/ml free CIP did not affect the survival rate of SCAPs in this study.

**Figure 5:**
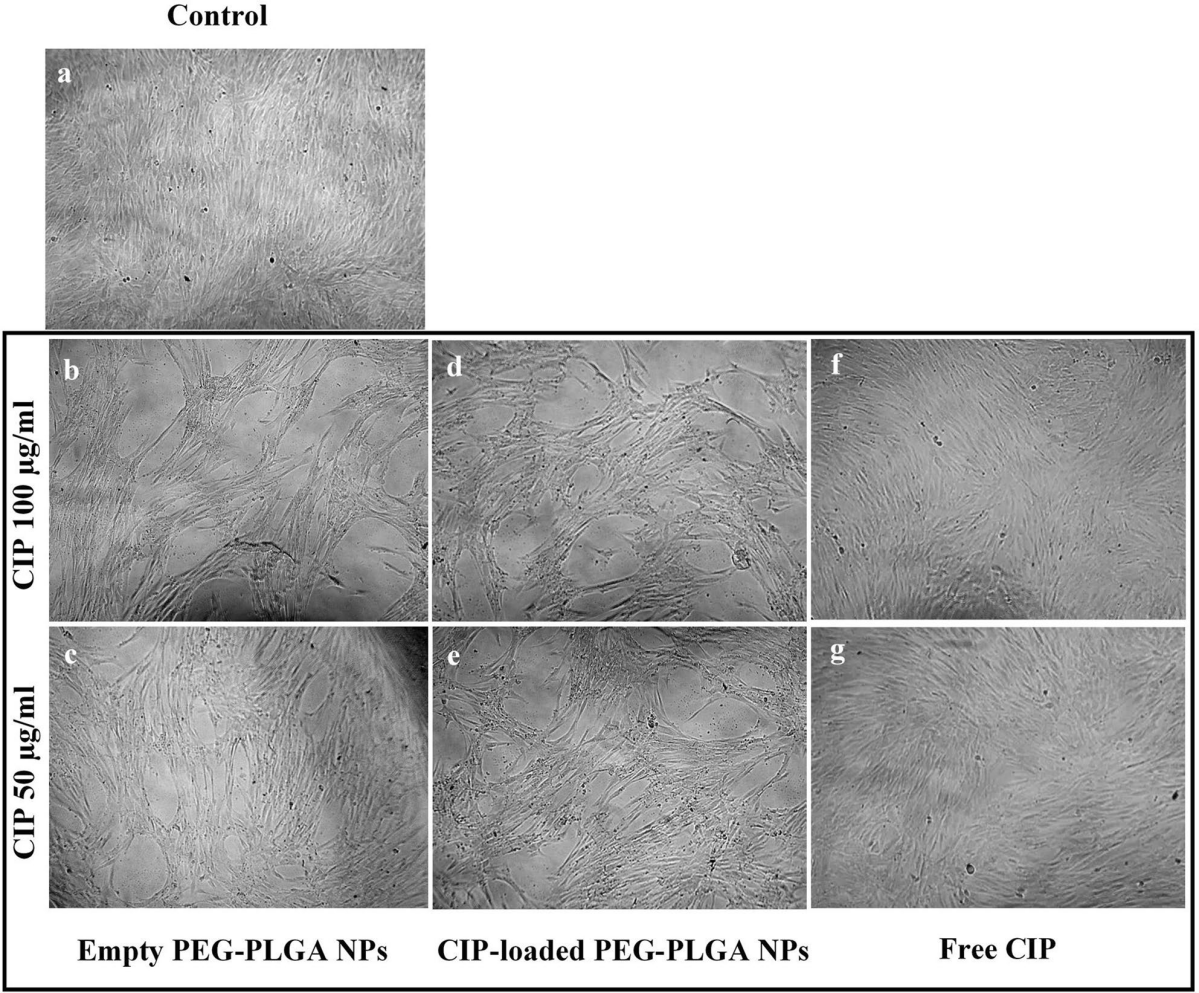
Stem cells from the apical papilla (SCAPs); (**A**) control (untreated cells) and after being treated with (**B–C**) empty PEG–PLGA nanoparticles, (**D–E**) CIP-loaded PEG–PLGA nanoparticles, and (**F–G**) free CIP for 24 h. The images were taken at 10X magnification using a Compact Cell Culture Microscope, CKX3 (Olympus).

The viability of the SCAPs treated with CIP-loaded PEG–PLGA NPs with 50 μg/ml and 100 μg/ml CIP were 86.65 (*P* ≤ 0.001) and 87.21% (*P* ≤ 0.0001), respectively, compared with untreated SCAPs (Fig. 4). The SCAP viability after being treated with CIP-loaded PEG–PLGA NPs at less than 10 μg/ml ciprofloxacin was found to be more than 95%, which similar to that of the untreated SCAPs (*P* > 0.05). Although a significant decrease in cell viability was observed, the percentages of cell viability were considered to be within the acceptable range (more than 70%) as described in ISO10993-5: 2009. Furthermore, lactic acid and glycolic acid, the byproducts of PLGA hydrolysis, might reduce the pH of the environment and affect the cell viability. However, dead cells were not observed in Fig. 5, and the viability of SCAPs treated with empty PEG–PLGA NPs was comparable with the control. These results suggest that the degradation of PLGA might not occur within 24 h and did not affect SCAP viability in this study. These results were also in agreement with a previous report^20^, in which human mesenchymal stem cells (hMSCs) treated with CIP-loaded PLGA and PCL NPs with 20 μg/ml CIP demonstrated ~ 80% cell viability. The results indicated that the CIP-loaded PEG–PLGA were compatible with SCAPs when applied in a range of 5–100 μg/ml CIP and provides an effective dose against *E. faecalis*.

The effect of sucrose (0.5–10%w/v) on SCAPs viability was also evaluated. The results revealed that the SCAP viability was not significantly different from the untreated SCAPs (see supplementary data, Fig. S2). 10%w/v sucrose was used to maintain and preserve the stability of the nanoparticles in the lyophilized powder (see supplementary data, Table S1) because this formulation is more convenient for long-term storage to maintain the stability and shelf-life of nanoparticles, increase the drug concentration, and prevent microorganism contamination^50^.

It should be noted that 2D SCAPs culturing slightly changed their morphology after empty PEG–PLGA NP (Fig. 5B–C) and CIP-loaded PEG–PLGA NP treatment (Fig. 5D–E) for 24 h, while the SCAPs treated with free CIP (Fig. 5F–G) appeared similar to the control cells (Fig. 5A). Dextran sulfate might affect the arrangement of the stem cells during culturing because it was used to prevent the aggregation of human pluripotent stem cells (hPSCs) in a 3D culture system^51^. Therefore, it could be possible that the dextran sulfate in the CIP-laded PEG–PLGA NPs might affect the intercellular interaction between cells, but not affect cell proliferation^51^. Another study used dextran sulfate (500 kDa) to enhance collagen I assembly and deposition in bone marrow mesenchymal stem cells (BMMSCs)^52,53^. Collagen I was reported to promote the initial attachment, survival, and stable growth of dental pulp stem cells in xenogeneic serum-free media, while maintaining their stemness^54^. Moreover, the addition of dextran sulfate enhanced the osteogenic differentiation rate in BMMSCs in osteogenic media^53,55^. Thus, the presence of dextran sulfate in the CIP-laded PEG–PLGA NPs might also promote the functional pulp regeneration by SCAPs in osteogenic conditions. These results suggest the possibility of using the antibiotic-loaded nanoparticles as a novel intracellular medicament to provide a clean and sterile environment during the tissue regeneration. Although there are many studies that have evaluated PEG–PLGA nanoparticles *in vivo*^56–58^, additional in vivo studies of CIP-loaded PEG–PLGA NPs should be performed in terms of their toxicity, long-term efficiency, and biological interaction with SCAPs.

## Conclusions

In summary, we successfully developed CIP-loaded PEG–PLGA NPs to be used in dental treatment. The NPs prepared using the S/O/W ion pairing technique demonstrated a spherical shape, good size distribution, and high CIP entrapment. The CIP-loaded PEG–PLGA NPs demonstrated an antibacterial effect against *E. faecalis* similar to that of free CIP. Furthermore, the NPs demonstrated low cytotoxicity to dental stem cells, suggesting that the NPs are biocompatible. Importantly, a significant improvement in CIP controlled release was achieved in this study. Taken together, CIP-loaded PEG–PLGA NPs exhibited desirable physical characteristics and effects as a topical antibiotic delivery system.

## Supplementary Information

Supplementary Information.

## Data Availability

The datasets generated during and/or analyzed during the current study are available from the corresponding author upon reasonable request.
