# Supplementary material for: PEG–PLGA nanoparticles for encapsulating ciprofloxacin
Source: Sci Rep. 2023 Jan 6;13:266. doi: 10.1038/s41598-023-27500-y (PMC9822989; doi:10.1038/s41598-023-27500-y)
Supplement: Supplementary file 1 — Supplementary Information. [file 41598_2023_27500_MOESM1_ESM.docx]

**Supplementary data for**

**PEG-PLGA Nanoparticles for Encapsulating Ciprofloxacin**

**Table S1. Characteristics of CIP-loaded PEG-PLGA nanoparticles after lyophilization with the addition of cryoprotectant.** The results reported as mean ± standard deviation (n = 3)

| Cryoprotectant | | Z-average (d.nm) | PdI |
| --- | --- | --- | --- |
| Name | %w/v |  |  |
| Control (Before lyophilization) | - | 104.8 ± 9.06 | 0.203 ± 0.040 |
| Control (After lyophilization) | - | Aggregates | Aggregates |
| Glucose | 5 | 203.2 ± 20.49 | 0.297 ± 0.036 |
| Sucrose | 5 | 154.7 ± 12.05 | 0.196 ± 0.012 |
| Sucrose | 10 | 100.14 ± 1.57 | 0.156 ± 0.006 |
| PEG 4000 | 5 | 246.3 ± 68.73 | 0.372 ± 0.070 |


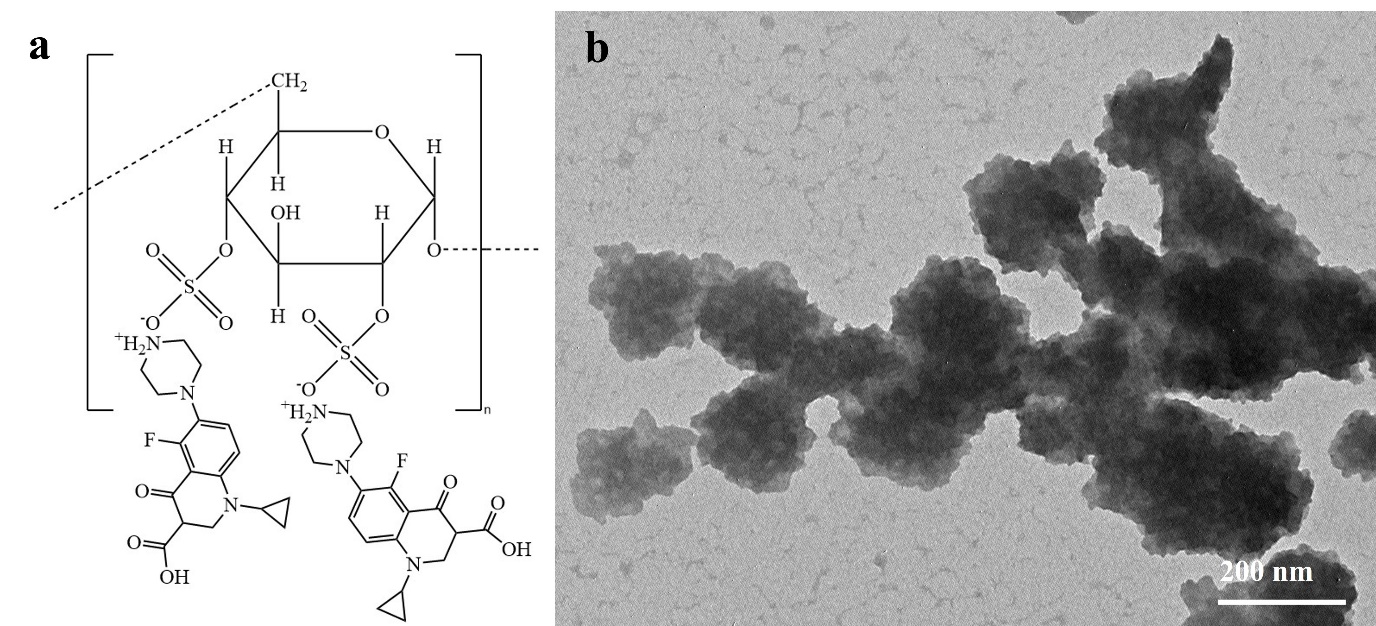


**Figure S1.** Proposed chemical structure of CIP-Dextran sulfate complexes formed via the electrostatic interaction (a) and electron micrograph of CIP-DS complexes observed under TEM at 20,000x magnification (b).


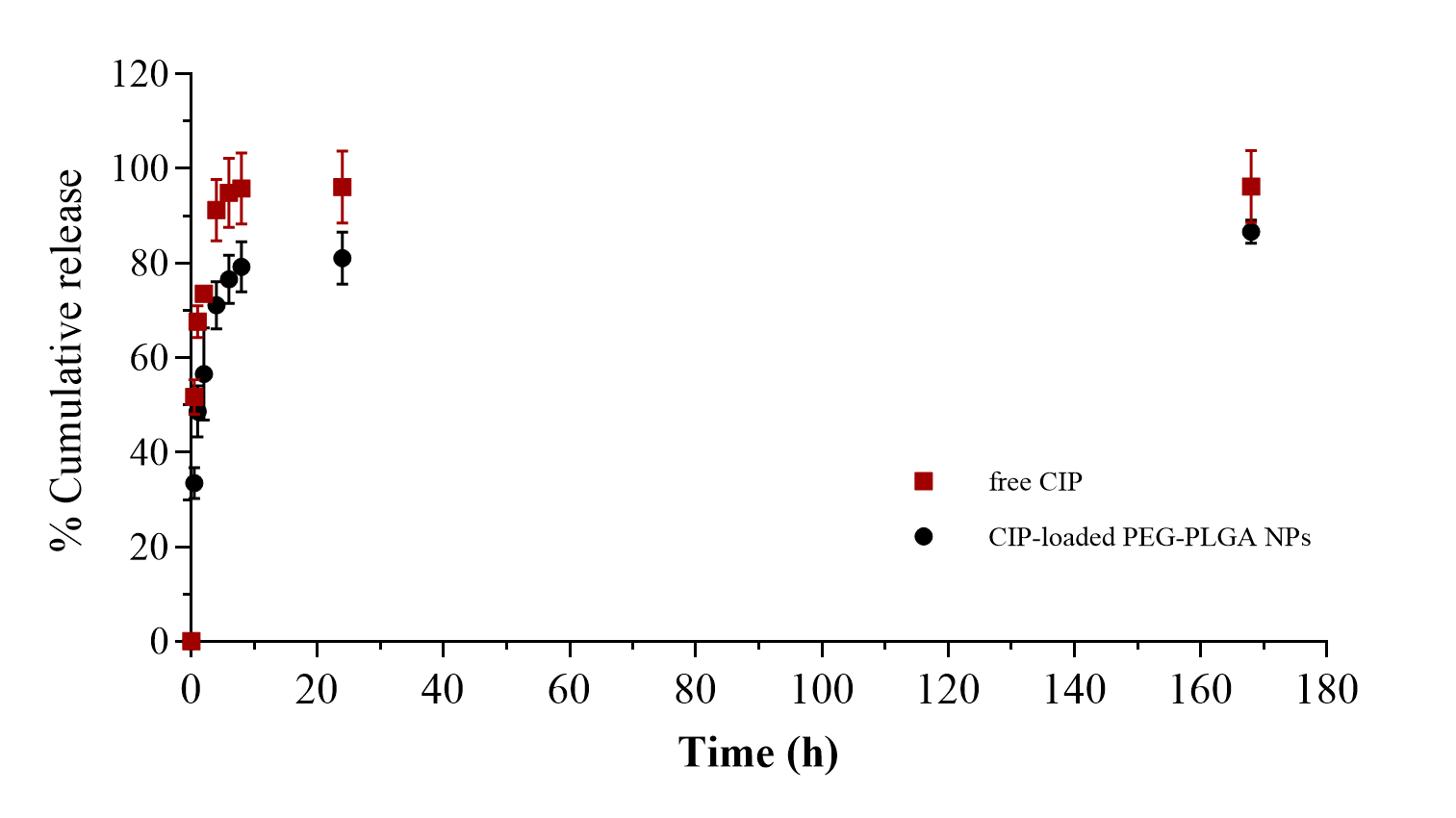


**Figure S2.** The cumulative release of free CIP (■) and CIP-loaded PEG-PLGA NPs (●) in tris-buffer saline solution, pH 7.4 during 168 h. Samples were shaken at the speed of 100 rpm at 37°C, under dark condition. The error bars in the plots represented the standard deviation obtained from three experimental replicates of each sample.

**
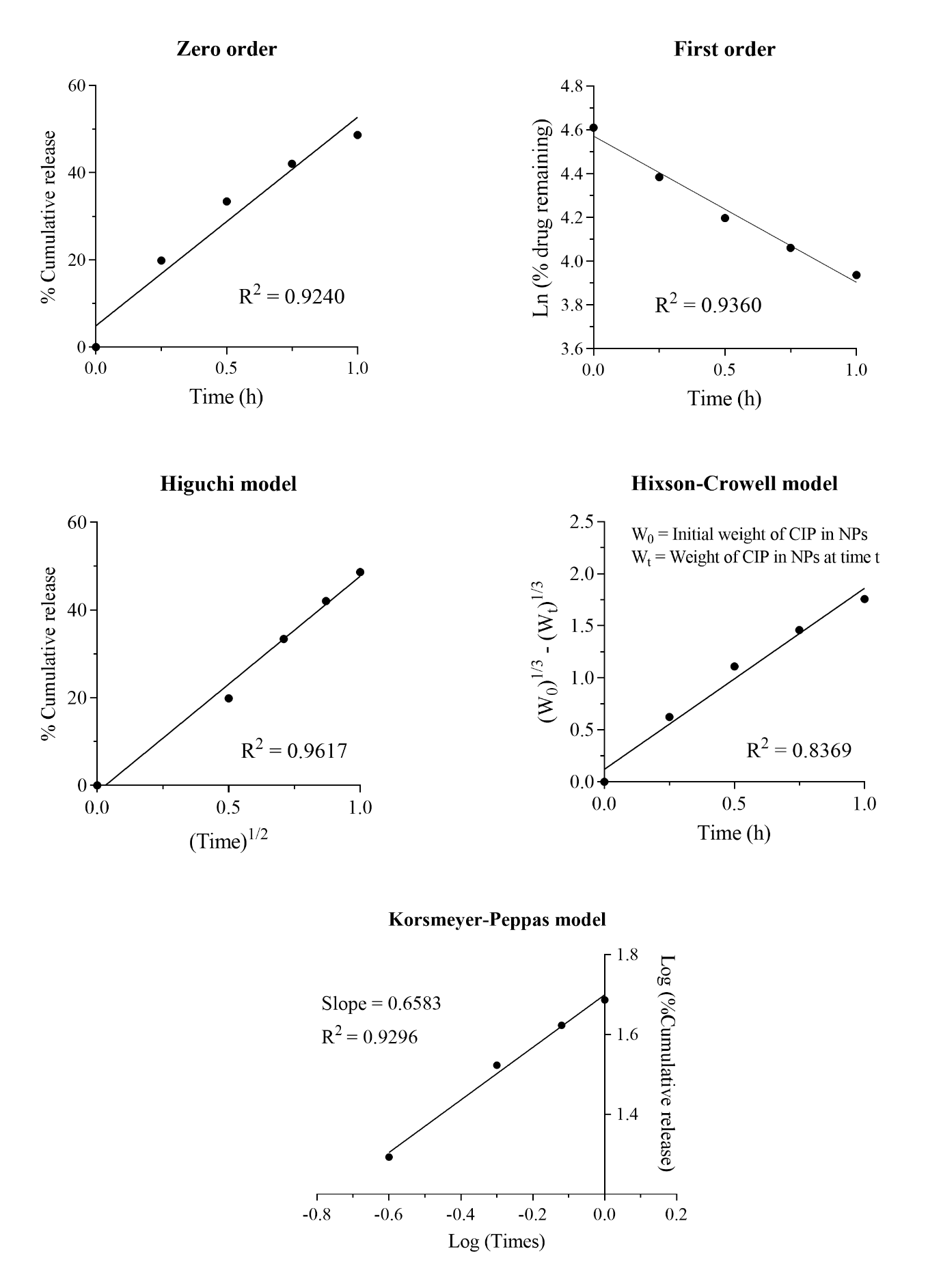
**

**Figure S3.** Kinetic analysis of ciprofloxacin released from PEG-PLGA nanoparticles using zero order, first order, Higuchi, Hixson-Crowell, and Korsmeyer-Peppas model. Data represented the average of three different samples.


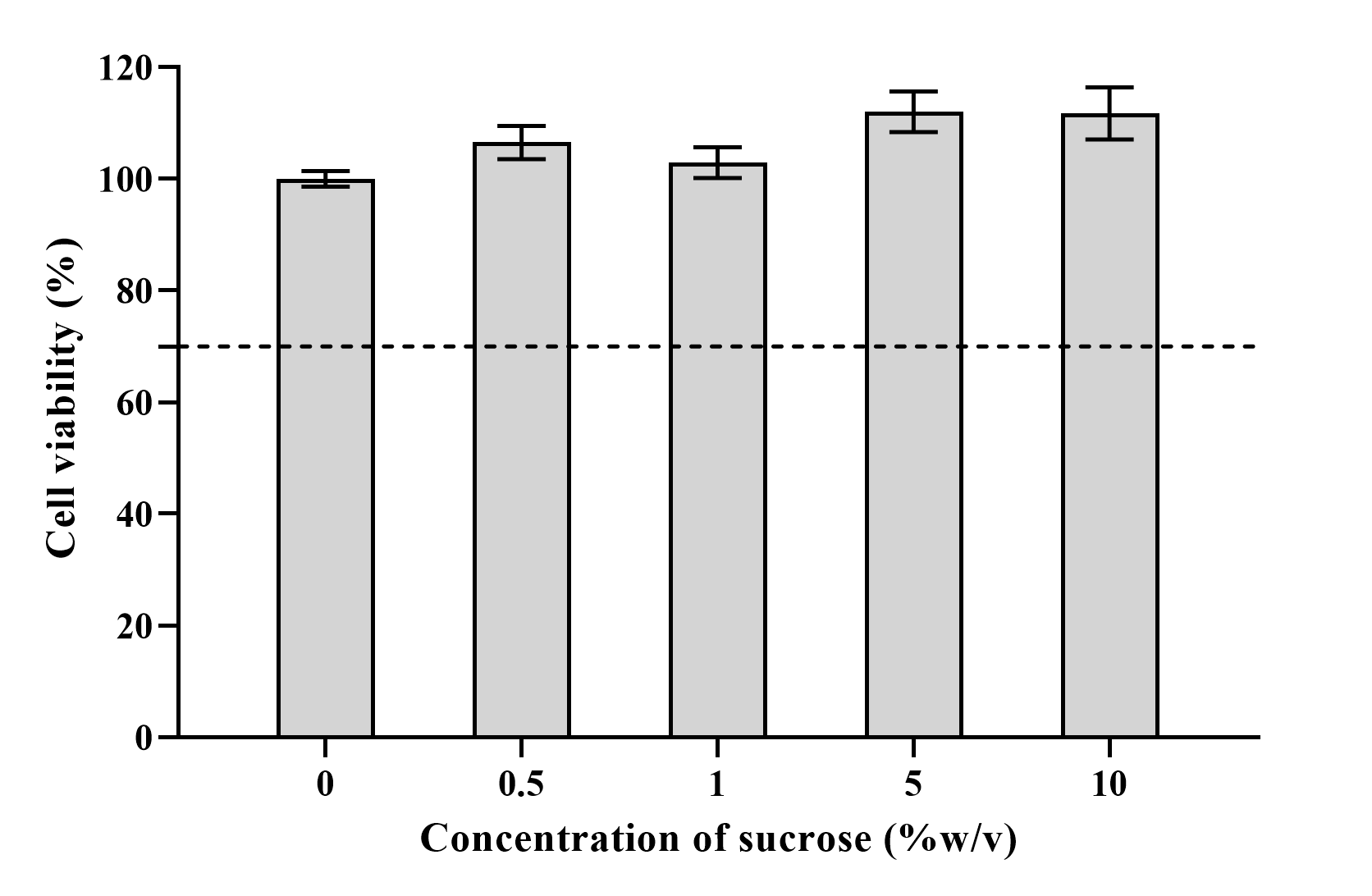


**Figure S4.** Cell viability of SCAPs after being treated with different concentrations of sucrose for 24 h, the error bars represented the standard deviation from three biological replications.
